# Immobilization of Cadmium by Fulvic Acid-Modified Palygorskite and Plant and Soil Metabolism Responses

**DOI:** 10.3390/toxics13020068

**Published:** 2025-01-21

**Authors:** Jianrui Li, Yingming Xu

**Affiliations:** 1 Department of Environmental and Safety Engineering, Taiyuan Institute of Technology, Taiyuan 030008, China; 2 Agro-Environmental Protection Institute, Ministry of Agriculture and Rural Affairs, Tianjin 300191, China; ymxu1999@126.com

**Keywords:** immobilization, cadmium, fulvic acid, modified palygorskite, metabolism responses

## Abstract

This experiment was designed to investigate the immobilization effect of fulvic acid-modified palygorskite on cadmium (Cd) and evaluate metabolism responses in plants in terms of chlorophyll, proline, and soluble protein and in soils in terms of microorganism number and enzymatic activity. The characteristics of the specific surface area and X-ray diffraction (XRD) spectra of modified palygorskite were analyzed to obtain information on the clay structure. The infrared (IR) spectrum characteristics of modified palygorskite and Cd adsorption products were analyzed to study the Cd immobilization mechanism. The modified palygorskite was hydrated magnesia aluminum silicate clay with a surface area of 50.923 m^2^/g and dominant mesopore distribution. The silanol group (Si-OH) and carboxyl (-COOH) present in modified palygorskite can form a complex with Cd to induce a 12.8–60.3% reduction in available Cd in soil and a 17.9–76.8% reduction in plant Cd. A 7.0–22.9% rise in chlorophyll, a 19.2–64.1% increase in proline, and a 20.1% maximum increase in soluble protein in plants were observed. A 1.45-fold maximal increase in number of bacteria, a 56.7% maximal rise in number of fungi, a 64.8–206.2% rise in dehydrogenase activity, and a 22.9-fold maximal increase in cellulase activity in the soil were obtained. Fulvic acid-modified palygorskite is a recommended Cd inactivator based on the fact that clay application reduces the ecological risk of Cd entering the food chain and stimulates plant physiological metabolism and soil biochemical activity.

## 1. Introduction

Farmland is a foundational resource in agricultural production, and the environmental quality of cultivated land has a decisive impact on the sustainable development of the economy and society. Heavy-metal pollution in agricultural land is a global concern for food security and human health. With the rapid advancement of industrialization in China, 19.4% of sampled farmland is polluted by heavy metals. Cadmium (Cd) is a heavy-metal element with strong mobility and biological toxicity, and the Cd contents in 36.1% of heavy-metal-contaminated farmland are higher than the Chinese soil environmental quality standard GB 15618-2018 limit of 0.30 mg/kg [1,2,3,4]. The heavy metal cadmium can damage the structure and function of plant cells, affect the permeability of cell membranes and the balance of ions within cells, and interfere with the absorption and distribution of nutrient elements in crops. The accumulation of Cd in the human body is due to Cd enrichment in the food chain, as well as Cd intake by hand, mouth, skin, and inhalation, resulting in functional and organic damage to multiple organs following Cd metabolism and transformation in organisms [5,6,7,8,9].

Health risk assessment and immobilization remediation techniques for heavy-metal pollution in cropland soils have become a focus in social research. Clay repair agents for metal pollution, with a high specific surface area, a pore size distribution of micropores and mesopores, and a negatively charged structural layer, have been applied for the immobilization remediation of Cd-polluted agricultural land in recent years [10,11,12]. Clay minerals are natural components of inorganic colloids in soils. Palygorskite is a hydrated magnesia aluminum silicate clay mineral with a layered chain structure, great cation exchange capacity, and good Cd adsorption performance. However, there needs to be an improvement in the capacity of adsorption of Cd on clay. Studies on the adsorption of heavy metals on modified clay have been carried out using a silane coupling agent and polyacrylamide as an organic modifier. Research has been conducted to increase the capacity of Cd to adsorb palygorskite by grafting sulfhydryl and amino groups on the surface of clay minerals [13,14,15,16]. It is widely known that the extensive use of organic modifiers may pose a risk of secondary pollution in soils. The application of environmentally friendly modifiers should be a main focus in further research. Fulvic acid is one of the natural components of soil humus and is rich in the oxygen-containing functional groups of carboxyl, hydroxyl, and carbonyl, making it a perfect selection for a modifier.

This pot experiment illuminated the effects of palygorskite and a fulvic acid-modified product on Cd availability in soils; Cd accumulation in vegetables; malondialdehyde (MDA), chlorophyll, proline, and soluble protein in plants; and microorganism numbers and enzymatic activity in soils to evaluate plant and soil metabolism responses under Cd-induced stress. The pH and the available contents of trace elements in treated soils were monitored to estimate the changes in physical and chemical properties. The BET (Brunauer, Emmett and Teller) surface area, pore size distribution, and X-ray diffraction (XRD) spectra of palygorskite and the modified product were characterized to obtain information on the clay structure. The infrared (IR) spectra of palygorskite, modified palygorskite, and the Cd adsorption product are characterized to explore the chemical pathway of Cd immobilization.

## 2. Materials and Methods

### 2.1. Palygorskite (PAL) and Fulvic Acid-Modified Product (PAL-C)

The palygorskite clay mineral adopted for Cd immobilization in polluted soils was obtained from Jiangsu Province in China and was a 2:1 hydrated magnesia aluminum silicate clay mineral with a layered chain structure. Fulvic acid (C_14_H_12_O_8_) was purchased from Biotechnology Co., Ltd., Changzhou, China. The pH value of fulvic acid was 5.31, achieved using the method provided by Lu [17].

Fulvic acid-modified palygorskite was prepared in a 1000 mL aqueous solution of 10.0 g of palygorskite mixed with 1.0 g of fulvic acid at 25.0 °C, which was stirred at a speed of 250 rpm (rotations per minute) for 24 h to complete the reaction between palygorskite and fulvic acid. The aqueous solution was then centrifuged at a speed of 2000 rpm to collect the solid phase, which was subsequently dried to constant weight at 70 °C to acquire fulvic acid-modified palygorskite. The pH values of palygorskite and the modified product were 9.26 and 8.83, respectively, with the method recommended by Lu [17].

### 2.2. Characterization of Soil Samples and Plant Cultures

The collection site for polluted soil samples was located in suburban farmland in Taiyuan city, Shanxi Province, China (112.57° E, 37.87° N), as shown in Supplementary Materials. The measured 2.03 mg/kg Cd accumulation in soils is mainly caused by long-term chemical fertilizer application and industrial activities. The physical and chemical properties of the soil samples were determined after passing them though a 0.85 mm sieve (20 meshes). Each soil sample was passed through a 0.15 mm sieve (100 meshes) to carry out the heavy-metal concentration test. The pH value and cation exchange capacity (CEC) of the soil samples before sowing were 6.06 and 121.8 mmol/kg, respectively, as obtained using the test methods provided by Lu [17]. The seeds of the tested cabbage plants (Jinqing No.3) were purchased from Shanxi Province in China. The average growth period of the plant is 70 d.

A topsoil sample from the vegetable field was collected at the soil depth of 0–20 cm. The soil sample was crushed to pass it through a 4 mm sieve (5 meshes). About 1.50 kg of the soil sample was placed in non-porous plastic pots. The palygorskite and fulvic acid-modified product clays were separately mixed with the Cd-polluted soil at the applied doses of 0, 5, 10, 15, 20, and 30 g/kg. Each treatment was performed in triplicate. After a 4-week interaction between soil and clay at 75.0% of the soil water-holding capacity, 4 vegetable seeds were planted in each plastic pot. Soil humidity was maintained with a supply of 400 g of tap water every two days during the plant growth period. The vegetable seedlings were harvested in July 2024 and washed with tap water (no Cd detected) after 10 weeks of growth. The above-ground plant sample was air-dried to a constant weight at 75 °C. The rhizosphere soil sample was collected by shaking off the soil attached to the roots. The plant sample was crushed with a stainless mill and passed through a 0.25 mm sieve for further analysis.

### 2.3. Analytical Methods

After plant harvest and soil sample collection, the pH value of the soil samples was tested using a pH meter (PHS-3C Lei-ci, Shanghai, China) in a ratio of water to solid of 2.0. The pH value of palygorskite was determined by using the same method used with the soil samples. The diluted hydrochloric acid (HCl) extraction method was adopted to determine the contents of available Cd in soils. About 5.0 g of soil from the sample was dispersed into 25 mL of 0.15 mol/L HCl solution and shaken for 60 min, and the Cd in the extracting solution was determined with an atomic absorption spectrometer (AA-6880 Shimadzu, Kyoto, Japan) to evaluate the content of available Cd in soils.

About 10.0 g of the soil sample was extracted by using 20 mL of mixed extraction solution composed of 0.005 mol/L DTPA (diethyltriamine pentaacetic acid), 0.01 mol/L CaCl_2_, and 0.1 mol/L TEA (triethanolamine), and, after 3000 rpm centrifugation, zinc (Zn) and copper (Cu) were detected in the supernatant by using the atomic absorption spectrometer (AA-6880 Shimadzu, Japan) to assess the available concentrations of Zn and Cu in polluted soils.

Plant and soil samples were digested by using solutions of HNO_3_ and HNO_3_–HF, respectively. The concentrations of Cd in the digestive solution were valued with an atomic absorption spectrometer (AA-6880 Shimadzu, Japan). Certified reference materials of polluted soil (GBW08303) and bush leaf (GBW08612) were utilized for quality control during the digestion process.

The characteristics of the BET specific surface area, pore size distribution, and X-ray diffraction (XRD) for palygorskite and the fulvic acid-modified product were determined to obtain information on the clay structure. The BET nitrogen (N_2_) absorption method was used to analyze the specific surface area and pore size distribution of palygorskite and the fulvic acid-modified product. The isothermal absorption experiments were conducted using an adsorption apparatus (Quantachrome, Autosorb iQ) with parameters including a N_2_ adsorption temperature of 77.350 k, a degassing temperature of 100 °C, an adsorption time of 1045.1 min, a degassing time of 60.0 min, a palygorskite weight of 0.3964 g in the sample tube, a N_2_ density of 0.808 g/cm^3^, a N_2_ molecular weight of 28.013 and a cross-sectional area of 16,200 Å^2^. The XRD patterns were recorded with a Bruker/D8 Advance X-ray diffractometer with Cu-Kα radiation, which was operated at 60 kV and 80 mA at a scanning speed of 2.0°/min over 2θ from 5° to 40°.

Isothermal experiments were carried out to investigate the adsorption effect of palygorskite and the fulvic acid-modified product on Cd^2+^ in CdCl_2_ solution at 25.0 °C. Palygorskite or the modified product weighing 1.00 g was passed through a 0.075 mm sieve and blended with 200 mL of CdCl_2_ solution with a series of Cd concentrations (C_0_) of 5, 10, 20, 30 and 50 mg/L; then, it was stirred at a speed of 250 rpm for 24 h to finish the isothermal adsorption experiment at pH 6.0. The Langmuir Equation was adopted to describe the pattern of adsorption of Cd on clay. Q_e_, Q_m_, C_e_, and b in the Langmuir Equation represent the equilibrium adsorption capacity, the maximum adsorption capacity, the equilibrium concentration, and a constant, respectively.

When the isothermal adsorption equilibrium is reached, the concentration of Cd^2+^ in a solution is called the equilibrium concentration (C_e_), and the Cd^2+^ adsorption capacity of clay is termed the equilibrium adsorption capacity (Q_e_). The maximum adsorption capacity (Q_m_), an important parameter for describing the chemical adsorption behavior on solid surfaces, refers to the maximum amount of Cd^2+^ that can be adsorbed by a unit of clay at Cd^2+^ concentrations of 5, 10, 20, 30, and 50 mg/L at 25 °C.

(1) $$Q_{e}=\frac{Q_{m}bC_{e}}{1+bC_{e}}$$

The concentrations of Cd in the supernatant after 3000 rpm centrifugation were determined with the atomic absorption spectrometer to calculate the equilibrium concentration (*C_e_*) of Cd in the adsorption solution and the Cd equilibrium adsorption capacity (Q*_e_*) of Cd on palygorskite and the modified product.

(2) $$Q_{e}=0.20\times {(C}_{0}-C_{e})$$

The Cd maximum adsorption capacity (Q_m_) on palygorskite and the modified product was determined based on the linear transformation of the Langmuir Equation.

(3) $$\frac{1}{Q_{e}}=\frac{1}{Q_{m}bC_{e}}+\frac{1}{Q_{m}}$$

The removal rate of Cd^2+^ in the adsorption solution (η) was calculated to estimate the Cd adsorption efficiency of Cd on palygorskite and the modified product.

(4) $$\eta =\frac{C_{0}-C_{e}}{C_{0}}$$

The infrared (IR) spectrum characteristics of palygorskite, fulvic acid-modified palygorskite, and the Cd adsorption product were determined to study the mechanisms behind Cd immobilization in palygorskite and the modified product. The infrared (IR) spectrum patterns with potassium bromide (KBr) tabletting were recorded with a Fourier transform infrared spectrometer (Nicolet NEXUS 670 ThermoFisher, Waltham, MA, USA) to analyze the oxygen-containing functional groups on the surface of palygorskite and the modified product, and a scanning time for each sample of 30 and a wave number range of 400–4000 cm^−1^ were adopted for sample characterization.

The changes in the concentrations of chlorophyll, proline, and soluble protein in cells reveal the plant physiological response to Cd stress in contaminated soils. An amount of 0.10 g of fresh leaves of the plants after harvest was immediately crushed in a mortar using liquid nitrogen and then stored at −80 °C in a fridge for preparing it for sample analysis. The determination of the contents of MDA (malondialdehyde), chlorophyll, proline, and soluble protein present in the abovementioned sample was carried out by using the corresponding Test Kit (Fantai, Shanghai, China). Dilution plate technology was used to determine the microorganism number in soils. A bacterium-mixing plate was utilized, 10^−5^–10^−7^ dilutions of the soil samples were used as inoculums, and the colonies were counted after culture at 30 °C for 3 days. A fungus-mixing plate was used, 10^−1^–10^−3^ dilutions of the samples were used as inoculums, and the colonies were determined after culture at 30 °C for 3 days [18]. The activity of dehydrogenase in soils was tested by using triphenyl tetrazolium chloride (TTC) as the substrate, with incubation at pH 7.6 and 37 °C for 24 h, and the triphenyl formazan (TPF) produced was determined with the colorimetric method. The activity of cellulase in soils was determined using carboxymethyl cellulose solution as the substrate, with incubation at pH 5.5 and 37 °C for 72 h, and the glucose produced was determined with the colorimetric method [19].

### 2.4. Data Analysis

All the experimental treatments were repeated three times. The means and standard deviations of the experimental data were estimated by using Microsoft Office Excel 2021. The analysis of the variance of repeated data and the correlation coefficient (R^2^) between data indicators were measured with Origin 2022 software. Multiple comparisons were performed by using the least significant difference (LSD) test when significant differences were observed among different treatments (*p* < 0.05). The significant differences between palygorskite and the modified product were analyzed by using the *t*-test (*p* < 0.05). The fittings of the Cd absorption isotherms were performed by using Origin 2022. Principal component analysis (PCA) for multiple variables was carried out with PCA 2017 software.

## 3. Results

### 3.1. Surface Area and Pore Size Distribution

**Figure 1:**
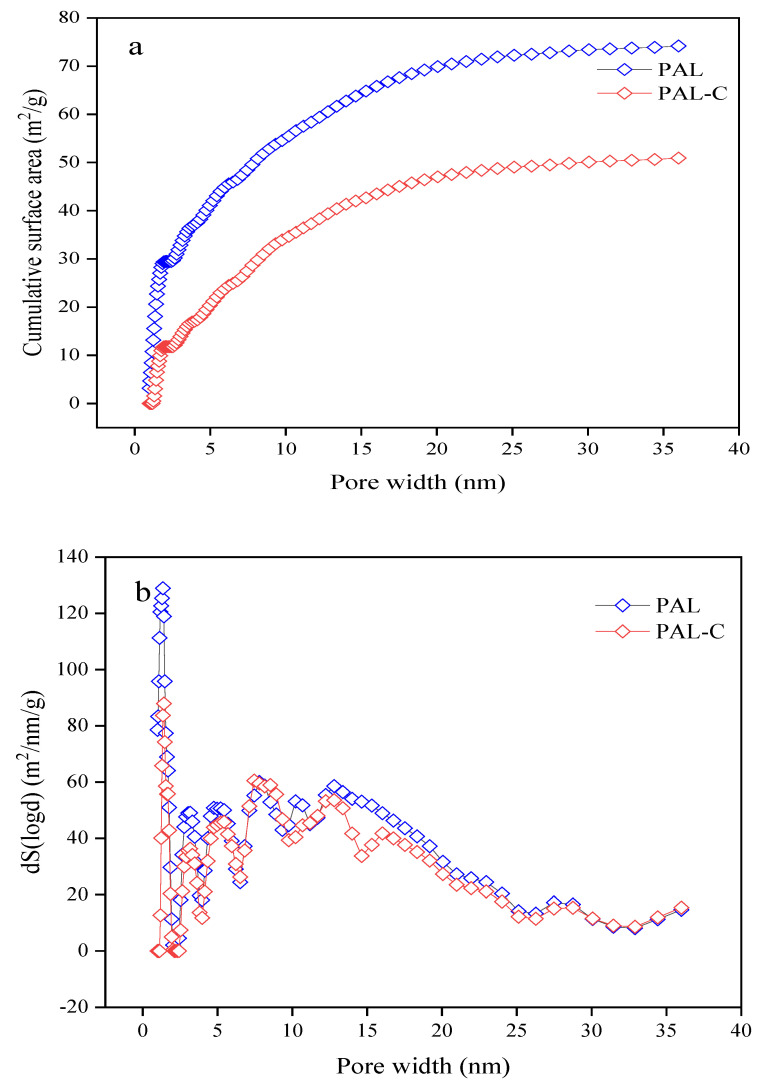
The specific surface area of palygorskite (PAL) and modified palygorskite (PAL−C). (**a**) Cumulative specific surface area; (**b**) change rate of specific surface area with respect to pore width.

The specific surface areas of palygorskite and the fulvic acid-modified product are shown in Figure 1. As reported in Figure 1a, the surface areas of palygorskite and the modified product are 74.185 m^2^/g and 50.923 m^2^/g, respectively, with a 31.4% reduction after modification (*p* < 0.05). The cumulative surface area of the micropores (0.9836–2.0208 nm) of palygorskite accounted for 39.7% of the totality and that of mesopores (2.0208–35.9996 nm) accounted for 60.3%. The surface area of the micropores of the modified product contributed 23.1% of the sum, and that of the mesopores contributed 76.9% (Figure 1b). The micropores of 0.9836–1.1776 nm in pore width were occupied by fulvic acid in the modified product, causing a significant reduction in the proportion of microporous surface area.

**Figure 2:**
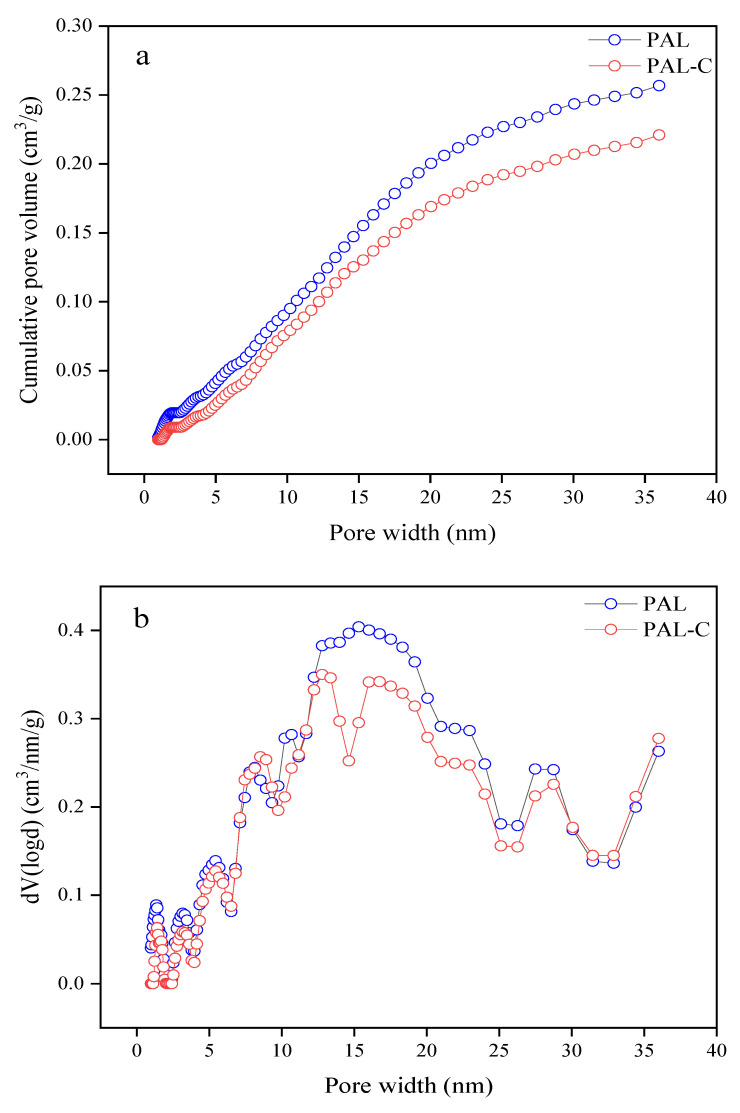
The pore size distribution of palygorskite (PAL) and modified palygorskite (PAL−C). (**a**) Cumulative pore volume; (**b**) change rate of pore volume with respect to pore width.

The pore size distributions of palygorskite and the modified product are shown in Figure 2. As reported in Figure 2a, the pore volume of palygorskite and the modified product are 0.25678 cm^3^/g and 0.22096 cm^3^/g, respectively, with a 13.9% decline due to the micropores being filled by fulvic acid during the modification process (*p* < 0.05). The pore volume of mesopores in palygorskite is 92.4% of the total volume, and that in the modified product is 96.0%, displaying the characteristics of a mesoporous material (Figure 2b).

### 3.2. XRD Spectra of Palygorskite and Modified Product

**Figure 3:**
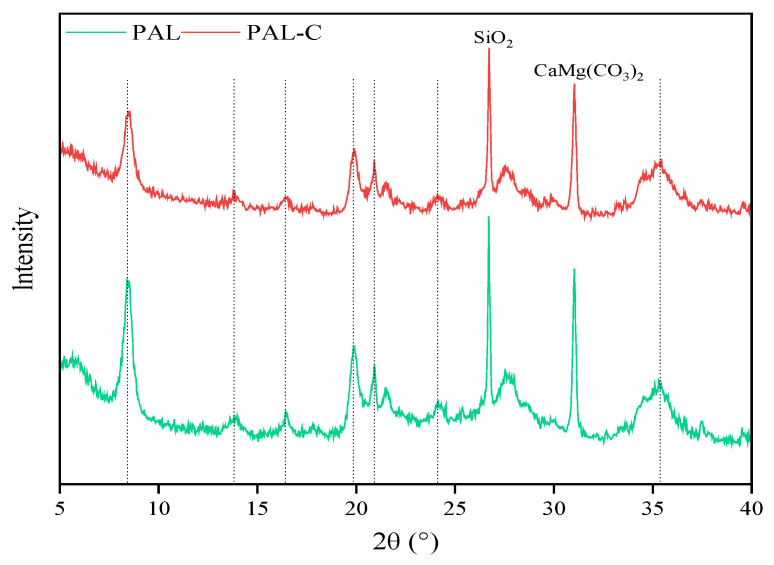
The spectra for X-ray diffraction (XRD) of palygorskite (PAL) and modified palygorskite (PAL−C).

The XRD patterns of palygorskite and the modified product are shown in Figure 3. The characteristic diffraction peaks of the crystal plane for palygorskite (Mg_5_(Si,Al)_8_O_20_(OH)_2_·8H_2_O, JCPDS No.31-0783) and the modified product are both found at 2θ = 8.39° (110 reflection). Differences in peak position are not found between palygorskite and the modified product, reflecting no changes in the structure characteristics of the latter. The impurity peaks of quartz and dolomite are synchronously detected at 2θ = 26.70° and 2θ = 31.08°, respectively. The XRD spectra reveal that the intensity of the diffraction peaks weakens in fulvic acid-modified palygorskite due to micropores being filled by the modifier on the surface of the clay, as revealed in Figure 1 and Figure 2.

### 3.3. Infrared Spectra of Palygorskite, Modified Palygorskite, and Cd Adsorption Product

**Figure 4:**
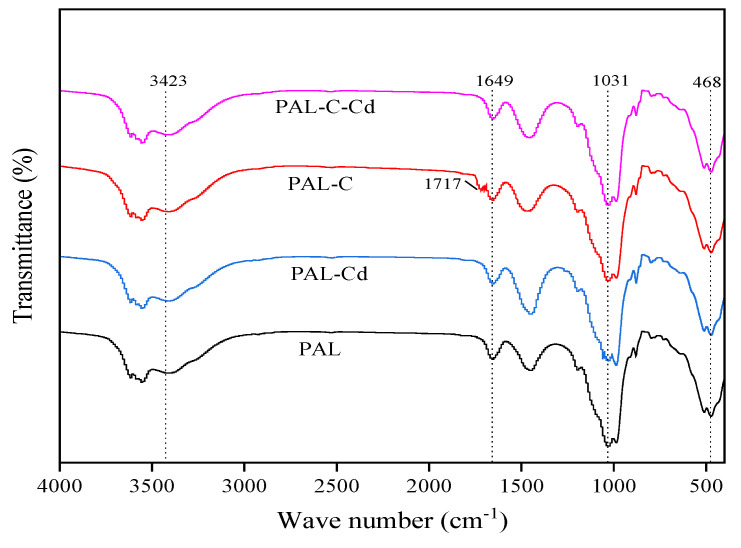
The infrared (IR) spectra of palygorskite (PAL), modified palygorskite (PAL−C), and Cd adsorption products (PAL−Cd and PAL−C−Cd).

The infrared spectra of palygorskite, modified palygorskite, and the Cd adsorption product are shown in Figure 4. Changes in absorption peak position are not observed among different treatments. The stretching vibration peak of the hydroxyl group in structural water (-OH), the bending vibration peak of the hydroxyl group in zeolitic water (H-O-H), and the stretching vibration peak of the silanol group (Si-OH) are detected at 3423 cm^−1^, 1649 cm^−1^, and 1031 cm^−1^. The new stretching vibration peak of carboxyl (-COOH) is found at 1717 cm^−1^ on the surface of modified palygorskite. The intensity of the vibration peak of the silanol group (Si-OH) is weaker in the Cd adsorption product than in palygorskite due to the formation of Cd(OH)_2_. The peak of carboxyl (-COOH) disappears after Cd absorption onto modified palygorskite owing to the complexation between Cd and carboxyl [20,21].

### 3.4. Cd Absorption by Palygorskite and Modified Product

**Figure 5:**
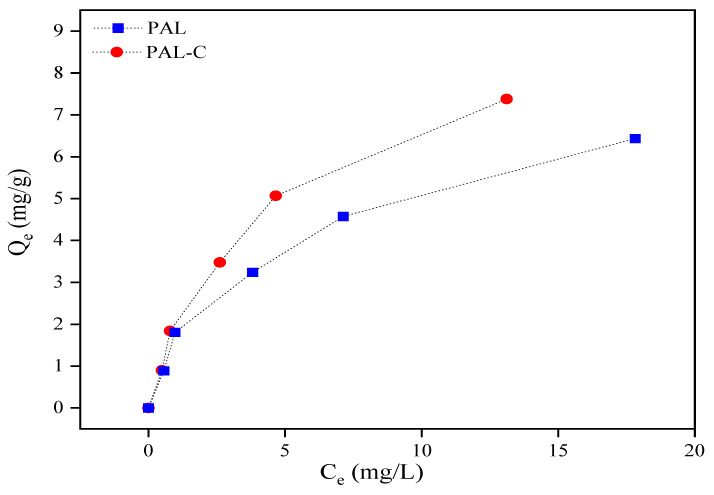
The isotherm adsorption of Cd on palygorskite (PAL) and modified palygorskite (PAL−C).

**Figure 6:**
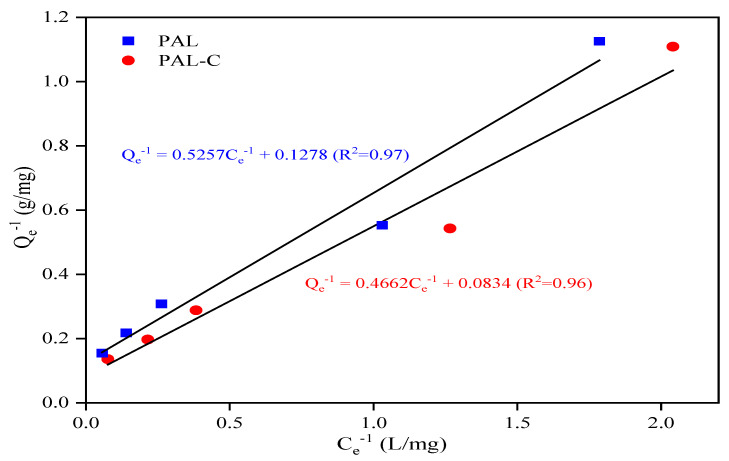
The Langmuir isotherm adsorption linear fitting of palygorskite (PAL) and modified palygorskite (PAL−C).

As shown in Figure 5, the palygorskite and modified product equilibrium isotherm data conform to the Langmuir Equation. The Cd equilibrium adsorption capacity, maximum adsorption capacity, equilibrium concentration, and constant in the Langmuir Equation are represented by Q_e_, Q_m_, C_e_, and b. The Cd removal rates (η) of 73.8–92.1% for the modified product in the aqueous environment are higher than the 64.4–90.3% rates for palygorskite at the initial Cd concentrations (C_0_) of 5, 10, 20, 30, and 50 mg/L. The linear fitting equation for the adsorption isotherm curve is shown in Figure 6; the Q_m_ values of palygorskite and the modified product are 7.82 mg/g and 11.99 mg/g, respectively, and the b values are 0.24 L/mg and 0.18 L/mg. The results for the Langmuir isotherm adsorption curve fitting reveal that the Cd adsorption on palygorskite and the modified product is mainly chemisorption. The specific surface area of the modified product is lower (Figure 1), but its Q_m_ is higher than that of palygorskite, indicating that the adsorption of Cd on the modified product is chemical adsorption rather than physical adsorption. The higher Q_m_ of the modified product is due to the silanol group (Si-OH) and carboxyl (-COOH) on the structure surface, which can form a complex with Cd^2+^ to form an insoluble compound.

### 3.5. Above-Ground Plant Biomass

**Figure 7:**
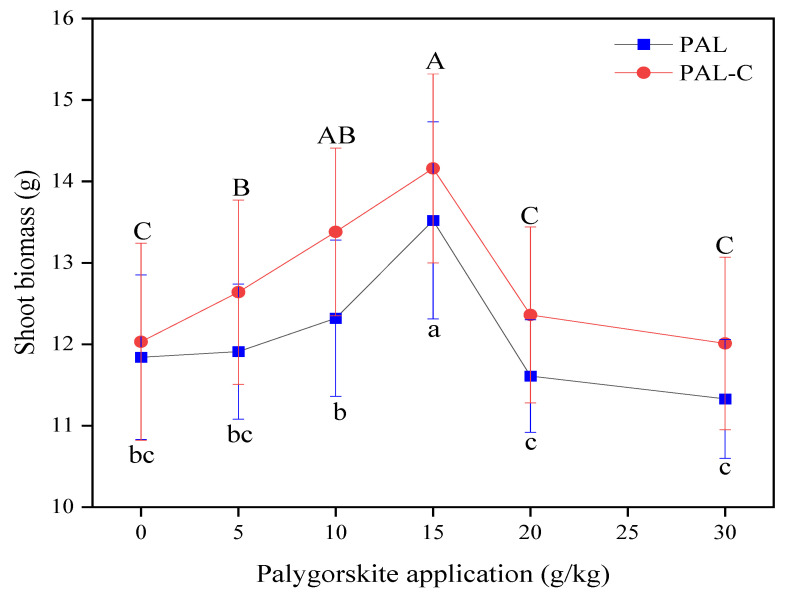
The biomasses (fresh weight) of shoots of plants grown in palygorskite (PAL)- and modified palygorskite (PAL−C)-treated soils. The data represented in the graph are means ± standard deviations. The means with different letters are significantly different for different clay applications (lowercase letter for PAL, uppercase letter for PAL−C) (n = 3, *p* < 0.05).

Excessive Cd in farmland delays seed germination and inhibits the elongation of plant roots and rhizomes, and above-ground biomass is a comprehensive indicator of plant growth status [22]. The shoot biomass in different treatments is shown in Figure 7. The 15 g/kg application of clay stimulates biomass accumulation, with maximum increases of 14.2% for palygorskite and 17.7% for the modified product compared with the no-clay-addition treatment (*p* < 0.05). There is a decline in biomass when the clay application is higher than 15 g/kg in soils. No significant differences are found among 0 g/kg, 20 g/kg, and 30 g/kg (*p* < 0.05). In the future, a rational clay application dose range should be recommended according to fluctuations in the above-ground plant biomass.

### 3.6. Cd Availability in Soils

**Figure 8:**
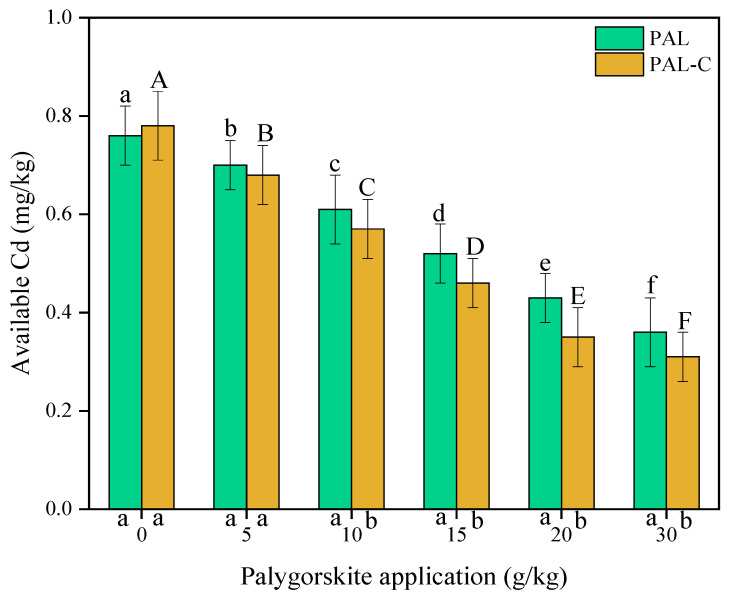
The contents of available Cd in palygorskite (PAL)- and modified palygorskite (PAL−C)-treated soils. The vertical bars represent means ± standard deviations. The different letters above bars are significantly different for different clay applications (lowercase letter for PAL, uppercase letter for PAL−C) (n = 3, *p* < 0.05). The different letters below the X axis are significantly different for PAL and PAL−C with the same clay application (*p* < 0.05).

The Cd absorption by palygorskite and the modified product plays an important role in reducing Cd availability in treated soils. As shown in Figure 8, the contents of available Cd in untreated soils are 0.76 mg/kg for palygorskite and 0.78 mg/kg for the modified product. The soil-available Cd decreases with the increase in the applied clay dose. The available Cd in palygorskite-treated soils reduces by 7.9–52.6% compared with treatment with no clay addition. The application of the modified product causes a 12.8–60.3% reduction (*p* < 0.05).

### 3.7. Concentrations of Cd in Plant Shoots

**Figure 9:**
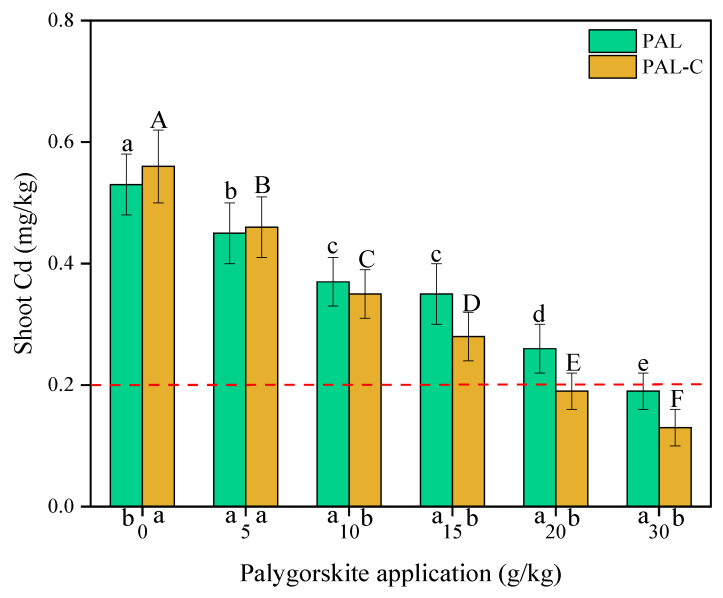
The concentrations of Cd in the shoots of plants grown in palygorskite (PAL)- and modified palygorskite (PAL−C)-treated soils. The vertical bars represent means ± standard deviations. The different letters above bars are significantly different for different clay applications (lowercase letter for PAL, uppercase letter for PAL−C) (n = 3, *p* < 0.05). The different letters below the X axis are significantly different for PAL and PAL−C with the same clay application (*p* < 0.05).

Cadmium uptake by plants depends on Cd availability in soils. As shown in Figure 9, compared with treatment with no clay, the concentrations of Cd in the shoots of plants decreases remarkably, with reductions of 15.1–64.2% for palygorskite and 17.9–76.8% for the modified product (*p* < 0.05). The addition of only 20 g/kg of the modified product leads to the Cd in the shoots being lower than the guideline value of 0.20 mg/kg, and the 30 g/kg palygorskite treatment meets the requirement of 0.20 mg/kg in the national food hygienic standard in China (GB2762-2012) [23]. Under the experimental condition, the addition of the modified product causes a 33.3% reduction in clay application in soils. Fulvic acid-modified palygorskite is a practicable solution in Cd pollution remediation, characterized by a lower applied dose of clay and a higher shoot biomass in vegetables (Figure 7).

### 3.8. Plant Physiological Status Under Cd-Induced Stress

Cadmium-induced oxidative stress in plants has received much attention in recent years. The contents of malondialdehyde (MDA), chlorophyll, proline, and soluble protein in the leaves of plants are monitored to explore the plant physiological metabolism mechanism under Cd stress, as shown in Table 1. The MDA is an indicator of cell membrane lipid peroxidation, reflecting the degree of damage to the cell membrane system [23]. In contrast to the treatment with no clay, the MDA in the leaves of plants decreases by 5.8–43.0% with palygorskite and 14.3–53.8% with the modified product, revealing the amelioration Cd-induced oxidative stress in plants (*p* < 0.05).

Chlorophyll is an important participant in plant photosynthesis, and the efficiency of photosynthesis can be gradually inhibited through a decrease in chlorophyll content in biological cells. After the addition of palygorskite and the modified product to soils, the contents of chlorophyll in leaves of plants increase by 9.8–24.5% and 7.0–22.9%, respectively, (*p* < 0.05). The proline in the leaves increases by 8.6–46.9% and 19.2–64.1% in the palygorskite and the modified product treatments (*p* < 0.05). The soluble protein in leaves maximally increases by 30.3% and 20.1% in the palygorskite and the modified product treatments (*p* < 0.05).

### 3.9. pH and Concentrations of Trace Elements in Soils

The pH value is one of the main limit factors for soil fertility and productivity, governing the species distribution and availability of trace elements in polluted soils. As outlined in Table 2, the pH in clay-treated soils rises significantly, with maximum increases of 0.67 units for palygorskite and 0.52 units for the modified product (*p* < 0.05). The basic oxides present in clay, including MgO, Al_2_O_3_, SiO_2_, K_2_O, and Na_2_O, cause these soil pH rises.

Trace elements are components of metabolic enzymes in plants, which are necessary for redox reactions, protein synthesis, photosynthesis, carbohydrate formation and migration, and reproductive organ development in plants. The available concentrations of Cu and Zn in the contaminated soils are outlined in Table 2. Overall, the extractable contents of Cu and Zn in palygorskite-treated soils decline with the increase in applied dose. The available Cu content in treated soils reduces by 13.8–33.9%, and palygorskite application reduces the available Zn by 7.5–20.8% (*p* < 0.05). The addition of modified palygorskite causes a 7.5–29.1% decline in available Cu and a 5.8–26.6% reduction in extractable Zn in treated soils (*p* < 0.05). Although palygorskite and the modified product treatments reduce the available contents of Cu and Zn in soils, the minimum concentrations are above the critical values of 1.5 mg/kg Zn and 2.0 mg/kg Cu for the normal growth and development of plants. In conclusion, the trace element supply is sufficient for the production of vegetables grown in treated soils.

### 3.10. Soil Biochemical Activity in Cd-Induced Stress

Microorganisms and enzymes are active components in soils. The number of microorganisms affects biochemical activity and nutrient composition and transformation. Bacteria and fungi are the main sources of microbial biomass, and microbial groups and quantity changes reveal the metabolism status of substances in soil. Soil microorganisms secrete various enzymes during their life processes, which participate in various biochemical reactions and play an important role in the formation of soil fertility and the material cycle in ecosystems. The complexation of Cd with the active centers of enzymes causes a decrease in enzymatic activity in soils. Heavy metals can affect soil enzyme activity by inhibiting microbial activity. Heavy metals can reduce the binding ability between microorganisms and substrates, leading to a decline in microbial quantity and activity [24,25,26,27].

As outlined in Table 3, the number of bacteria in the palygorskite- and modified product-treated soils maximally increases by 1.31 times and 1.45 times, and soil fungi rise by 52.3% and 56.7%, respectively (*p* < 0.05). The dehydrogenase activity in soil in the palygorskite and modified product treatments increases by 54.2–20.48% and 64.8–206.2%, and cellulase activity rises by 16.9 times and 22.9 times maximally (*p* < 0.05). The soil biological activity and the fertility index are remarkably improved.

## 4. Discussion

It would be practical to employ fulvic acid-modified palygorskite for Cd-contaminated soil remediation. Fulvic acid-modified palygorskite, rich in silanol groups (Si-OH) and carboxyl (-COOH), has a Cd maximum adsorption capacity (Q_m_) of 11.99 mg/g, and the Q_m_ of palygorskite is only 65.2% that of the modified product, as shown in Figure 6. In a study on the adsorption of Cd^2+^ on mercapto- and amino-functionalized palygorskite, thiol (-SH) and amino (-NH_2_) groups were grafted onto the surface of a palygorskite structure by using 3-mercaptopropyltrimethoxysilane and 3-aminopropyltrimethoxysilane as modifiers, respectively. The Q_m_ value of pristine palygorskite was 33.6% of that of mercapto-modified palygorskite and 42.2% of that of amino-functionalized palygorskite [13]. Although a perfect adsorption effect of Cd on modified palygorskite was achieved in the above-mentioned research study, the potential harmful effects of silane coupling agents in soil and aqueous environments should not be ignored, considering sustainable development strategies and the future of civilization. In general, the introduction of new pollutants is not advisable in soil contamination remediation.

The biomass of the above-ground parts of the plants in the treated soils rises significantly, as shown in Figure 7. Besides bioavailable Cd reduction, the changes in soil enzymatic activities and the microbial numbers of the soil fertility index in treated soils may represent another reason behind biomass increase [28,29]. Similar results were also obtained in this study. The greater Cd immobilization ability of the modified product compared with palygorskite is the principal reason for there being less available Cd in the treated soils. Clay mineral application gives rise to notable increases in Cd residual and oxide-bound fractions and a reduction in extractable Cd in the contaminated soils, and the Cd ecological risk clearly declines in the farmland ecosystem [30,31].

A decrease in the concentration of Cd in plants and an increase in plant biomass are observed after applying modified palygorskite to contaminated soil, as shown in Figure 7 and Figure 9. A study on the effect of the addition of thiolated palygorskite to soil on Cd accumulation in plants revealed that mercapto-functionalized palygorskite could significantly reduce Cd concentrations and the subcellular distribution of Cd in plants. The above-ground biomass of plants increased after the application of modified clay to polluted soils [12]. Thiolated palygorskite application caused a decline in the content of phytoavailable Cd in soils, a reduction in the biological concentration factor of three cultivars of leafy vegetables, and a decrease in Cd accumulation in the roots and shoots of pakchoi and romaine lettuce grown in Cd-polluted soils [32].

The weaker oxidative cell damage characterized by a lower MDA content in the leaves of plants grown in clay-treated soils could be attributed to higher activities of antioxidant enzymes in cells, including superoxide dismutase (SOD), peroxidase (POD), and catalase (CAT). SOD can eliminate oxygen free radicals (O_2_^−^) through the disproportionation reaction of O_2_^−^ to H_2_O_2_, and POD and CAT can promote the decomposition of H_2_O_2_ to produce H_2_O [11]. The remarkable increases in chlorophyll and osmotic-adjusting materials in the biological cells of plants grown in treated soils (Table 1) indicate the improvement of plant growing conditions, which is confirmed by the rise in the above-ground biomass of plants. The lower concentrations of Cd in plants grown in the treated soils reduce the oxidative stress induced by Cd in biological metabolism and stimulate plant protection mechanisms. Osmotic-adjusting materials including proline and soluble protein can maintain a normal physiological metabolism in plants by increasing the quantity of functional proteins. The plant cell can construct physical barriers by regulating osmotic pressure to alleviate Cd oxidative stress [33]. Moreover, the decline in chlorophyllase activity induced by Cd accumulation reduction in plants promotes chlorophyll formation [34].

**Figure 10:**
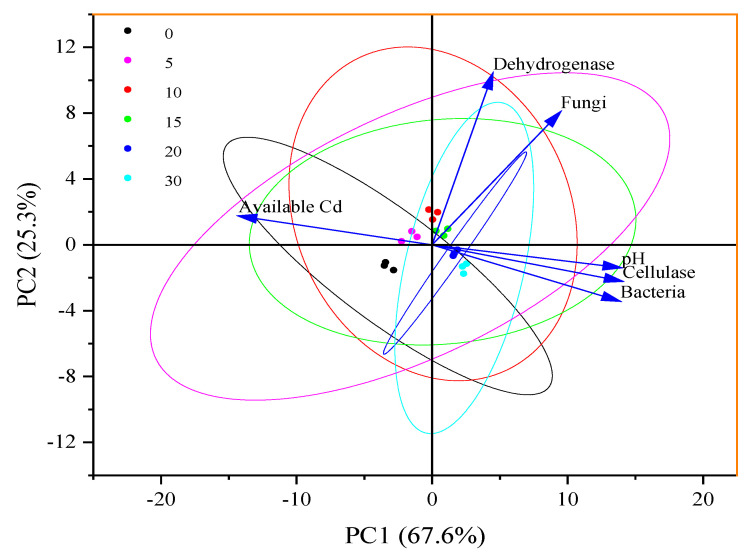
Principal component analysis for pH, available Cd, bacteria, fungi, dehydrogenase, and cellulase in soils. PC1 and PC2 represent the first and second principal components, respectively.

In addition, as shown in Table 3, significant rises in the number of bacteria and fungi in palygorskite- and modified product-treated soils are observed, and the activities of enzymes in treated soils, including dehydrogenase and cellulase, rise after clay application. As shown in Figure 10 and Supplementary Materials, principal component analysis for pH, available Cd, bacteria, fungi, dehydrogenase, and cellulase in soils was performed to reveal the relationships among multiple variables. The variance contribution rate (Proportion) of the first and second principal components are 67.6% and 25.3%, respectively, resulting in a cumulative Proportion of 92.9%. The pH value is positively related with bacteria number (R^2^ = 0.89) and cellulase activity (R^2^ = 0.84) in soils (*p* < 0.05), and available Cd is negatively related with the number of bacteria number (R^2^ = 0.91) and cellulase activity (R^2^ = 0.93) in soils (*p* < 0.05). pH increases cause increases in the number of bacteria and cellulase activity. Fungi have a higher tolerance to heavy metals than bacteria and an optimum pH range of 4.0–6.0, leading to these organisms being less sensitive to pH rises and available Cd reductions. Instead, a pH rise retards fungus proliferation in soils when exceeding 6.50 (Table 2). The reduction in available Cd in clay-treated soils alleviates the oxidative stress induced by Cd in microorganisms and enzymes. Furthermore, microorganisms with anionic groups can combine with positively charged Cd^2+^ in soil solutions, altering the chemical form, distribution, and biological toxicity of metal pollutants [28,35]. As we expected, the decline in available Zn and Cu contents in treated soils has no adverse influence on plant growth and development.

## 5. Conclusions

Mesoporous material fulvic acid-modified palygorskite is an ideal candidate as a remediation material to be used in Cd-polluted vegetable soils. A Cd maximum adsorption capacity of 11.99 mg/g for modified palygorskite was obtained in the isothermal adsorption test. The silanol group (Si-OH) and carboxyl (-COOH) present in modified palygorskite could form a complex with Cd to reduce the content of available Cd in treated soils and the accumulation of Cd in the above-ground parts of plants. A 20 g/kg clay application in soils with 2.03 mg/kg Cd meant that the concentration of Cd in the shoots was lower than the guideline of 0.20 mg/kg set by the food safety standard in China (GB2762-2012) [23]. The contents of chlorophyll, proline, and soluble protein in plant after modified palygorskite addition rose remarkably to reflect the improvement in plants’ physiological metabolism status. The remarkable increases in the microorganism number and enzymatic activity revealed the recovery of biochemical activity and the fertility index in the soil ecosystem. The supply of trace elements in treated soils meet the requirements for plant growth.

## Figures and Tables

**Table 1:** The contents of malondialdehyde, chlorophyll, proline, and soluble protein in plants.

Clay Dose (g/kg)	Malondialdehyde (μmol/g)		Chlorophyll (mg/kg)		Proline (nmol/g)		Soluble protein (nmol/mg)	
	PAL	PAL−C	PAL	PAL−C	PAL	PAL−C	PAL	PAL−C
0	0.086 ± 0.006 a (a)	0.091 ± 0.008 a (a)	1.63 ± 0.11 c (a)	1.57 ± 0.13 d (a)	0.81 ± 0.06 d (a)	0.78 ± 0.06 d (a)	0.152 ± 0.02 d (a)	0.159 ± 0.02 d (a)
5	0.081 ± 0.007 a (a)	0.078 ± 0.009 ab (a)	1.79 ± 0.13 bc (a)	1.68 ± 0.16 c (a)	0.88 ± 0.05 cd (a)	0.93 ± 0.08 c (a)	0.166 ± 0.01 c (a)	0.166 ± 0.03 c (a)
10	0.069 ± 0.005 b (a)	0.066 ± 0.007 b (a)	1.86 ± 0.21 b (a)	1.79 ± 0.13 b (a)	0.93 ± 0.07 c (a)	1.06 ± 0.11 b (a)	0.169 ± 0.02 bc (a)	0.169 ± 0.02 c (a)
15	0.062 ± 0.006 b (a)	0.063 ± 0.006 b (a)	1.88 ± 0.17 b (a)	1.81 ± 0.21 b (a)	1.02 ± 0.11 b (a)	1.08 ± 0.13 b (a)	0.173 ± 0.02 b (a)	0.178 ± 0.02 b (a)
20	0.055 ± 0.006 c (a)	0.053 ± 0.005 c (a)	1.95 ± 0.22 ab (a)	1.92 ± 0.18 a (a)	1.08 ± 0.09 b (a)	1.19 ± 0.09 ab (a)	0.196 ± 0.02 a (a)	0.183 ± 0.01 ab (a)
30	0.049 ± 0.005 c (a)	0.042 ± 0.003 d (a)	2.03 ± 0.19 a (a)	1.93 ± 0.16 a (a)	1.19 ± 0.12 a (a)	1.28 ± 0.13 a (a)	0.198 ± 0.03 a (a)	0.191 ± 0.03 a (a)

All data in the Table represent means ± standard deviations. Different letters in the column are significantly different at different clay doses (*p* < 0.05). Letter enclosed in parentheses in each row is significantly different between PAL and PAL−C (*p* < 0.05). PAL, palygorskite; PAL−C, modified palygorskite.

**Table 2:** The pH value and concentrations of available Cu and available Zn in soils.

Clay Dose (g/kg)	pH		Available Cu (mg/kg)		Available Zn (mg/kg)	
	PAL	PAL−C	PAL	PAL−C	PAL	PAL−C
0	6.22 ± 0.11 d (a)	6.21 ± 0.13 d (a)	21.8 ± 0.91 a (a)	21.3 ± 0.86 a (a)	56.3 ± 2.21 a (a)	57.1 ± 1.86 a (a)
5	6.34 ± 0.12 cd (a)	6.31 ± 0.11 c (a)	18.8 ± 0.83 b (b)	19.7 ± 0.77 b (a)	52.1 ± 2.06 b (a)	53.8 ± 1.91 b (a)
10	6.48 ± 0.07 c (a)	6.44 ± 0.06 bc (a)	17.7 ± 0.72 c (b)	18.9 ± 0.66 c (a)	48.2 ± 2.08 c (a)	49.9 ± 2.11 c (a)
15	6.63 ± 0.11 b (a)	6.53 ± 0.12 b (a)	17.9 ± 0.68 c (b)	18.8 ± 0.61 c (a)	47.7 ± 1.61 c (a)	46.1 ± 1.38 d (a)
20	6.72 ± 0.09 ab (a)	6.59 ± 0.07 ab (b)	15.2 ± 0.66 d (a)	15.5 ± 0.69 d (a)	45.1 ± 1.33 d (a)	45.7 ± 1.66 d (a)
30	6.89 ± 0.13 a (a)	6.73 ± 0.08 a (b)	14.4 ± 0.52 e (b)	15.1 ± 0.51 d (a)	44.6 ± 1.82 d (a)	41.9 ± 1.91 e (b)

All data in the Table represent means ± standard deviations. Different letters in the column are significantly different at different clay doses (*p* < 0.05). Different letters enclosed in parentheses on each row are significantly different between PAL and PAL−C (*p* < 0.05). PAL, palygorskite; PAL−C, modified palygorskite.

**Table 3:** The microorganism number and enzymatic activity in soils.

Clay Dose (g/kg)	Bacteria (10^7^ CFU/g)		Fungi (10^5^ CFU/g)		Dehydrogenase (mg TPF/g/h)		Cellulase (μmol Glucose/g/h)	
	PAL	PAL−C	PAL	PAL−C	PAL	PAL−C	PAL	PAL−C
0	1.21 ± 0.11 e (a)	1.27 ± 0.09 f (a)	2.87 ± 0.23 d (a)	2.75 ± 0.18 c (a)	16.8 ± 0.93 e (a)	16.2 ± 0.81 d (a)	0.015 ± 0.01 f (a)	0.013 ± 0.01 f (a)
5	1.44 ± 0.08 d (a)	1.41 ± 0.11 e (a)	3.51 ± 0.33 bc (a)	3.58 ± 0.31 b (a)	33.8 ± 1.82 bc (a)	34.7 ± 1.66 bc (a)	0.086 ± 0.01 e (a)	0.095 ± 0.01 e (a)
10	1.77 ± 0.13 c (a)	1.83 ± 0.08 d (a)	4.37 ± 0.38 a (a)	4.31 ± 0.33 a (a)	51.2 ± 2.61 a (a)	49.6 ± 2.38 a (a)	0.141 ± 0.01 d (a)	0.143 ± 0.01 d (a)
15	2.03 ± 0.09 b (a)	2.15 ± 0.13 c (a)	4.11 ± 0.35 ab (a)	4.26 ± 0.26 a (a)	37.2 ± 1.35 b (a)	38.8 ± 1.21 b (a)	0.163 ± 0.02 c (a)	0.179 ± 0.02 c (a)
20	2.37 ± 0.16 ab (b)	2.58 ± 0.14 b (a)	3.87 ± 0.31 b (b)	4.03 ± 0.28 ab (a)	30.8 ± 1.56 c (a)	29.3 ± 1.47 c (a)	0.252 ± 0.03 b (a)	0.281 ± 0.04 b (a)
30	2.79 ± 0.21 a (b)	3.11 ± 0.23 a (a)	3.21 ± 0.26 c (b)	3.63 ± 0.30 b (a)	25.9 ± 1.41 d (a)	26.7 ± 1.19 cd (a)	0.268 ± 0.03 a (b)	0.311 ± 0.04 a (a)

All data in the Table represent means ± standard deviations. Different letters in the column are significantly different (*p* < 0.05). Different letters enclosed in parentheses on each row are significantly different between PAL and PAL−C (*p* < 0.05). PAL, palygorskite; PAL−C, modified palygorskite. TPF, triphenyl formazan.

## Data Availability

All data generated or analyzed in this study are included in the article.

## Supplementary Materials

The following supporting information can be downloaded at: https://www.mdpi.com/article/10.3390/toxics13020068/s1, Figure S1. Map of provincial cities of China; Figure S2. Principal component analysis.
